# Synthesis of a New Molecularly Imprinted Polymer and Optimisation of Phenylglyoxylic Acid Extraction from Human Urine Samples Using a Central Composite Design within the Response Surface Methodology

**DOI:** 10.3390/polym15153279

**Published:** 2023-08-02

**Authors:** Murad. M. Qronfla, Bassem Jamoussi, Radhouane Chakroun, Bandar A. Al-Mur, Riyadh F. Halawani, Fahed A. Aloufi

**Affiliations:** Department of Environment, Faculty of Environmental Sciences, King Abdulaziz University, Jeddah 21589, Saudi Arabia; muradqronfla@yahoo.com (M.M.Q.); rshagroon@kau.edu.sa (R.C.); balmur@kau.edu.sa (B.A.A.-M.); rhalawani@kau.edu.sa (R.F.H.); faloufi@kau.edu.sa (F.A.A.)

**Keywords:** molecularly imprinted polymer (MIP), solid phase extraction (SPE), urine sample, phenylglyoxylic acid (PGA), central composite design (CCD), response surface methodology (RSM)

## Abstract

Styrene, a chemical widely used in various industries, undergoes metabolic breakdown in the human body, resulting in the production of phenylglyoxylic acid (PGA). A novel molecularly imprinted polymer (MIP) was synthesised for selective extraction and enrichment of PGA in urine samples prior to high-performance liquid chromatography. The MIP employed in this research was a 4-vinylpyridine molecularly imprinted polymer (4-VPMIP) prepared via mass polymerisation using a noncovalent method. The structural and morphological characteristics of the molecularly imprinted polymers (MIPs) and non-imprinted polymers (NIPs) were evaluated using Fourier transform infrared spectroscopy (FT-IR) and scanning electron microscopy (SEM). The efficiency of the molecularly imprinted solid-phase extraction (MISPE) process was optimised by investigating critical variables such as sample pH, sorbent mass, sample flow rate, and volume of the elution solvent. A central composite design (CCD) within the response surface methodology was utilised to develop separate models for the adsorption and desorption steps. Analysis of variance (ANOVA) confirmed the excellent fit of the experimental data to the proposed response models. Under the optimised conditions, the molecularly imprinted polymers exhibited a higher degree of selectivity and affinity for PGA, with a relative selectivity coefficient (α) of 2.79 against hippuric acid. The limits of detection (LOD) and quantification (LOQ) for PGA were determined to be 0.5 mg/L and 1.6 mg/L, respectively. The recoveries of PGA ranged from 97.32% to 99.06%, with a relative standard deviation (RSD) lower than 4.6%. Furthermore, MIP(4VP)SPE demonstrated the potential for recycling up to three times without significant loss in analyte recovery.

## 1. Introduction

Given the growing concerns about dangerous chemicals such as styrene in the environment and workplace, it is important to keep an eye on toxic chemical compounds and their metabolites, to monitor the risks they represent and what obstacles they may bring [1]. The chemical compound known as styrene (or vinyl-benzene) is a crucial component in various industrial applications, particularly in the production of plastics and rubber [2,3]. However, individuals employed in manufacturing processes are susceptible to the detrimental effects of volatile organic compounds (VOCs), which pose a significant risk to the health of workers and have negative environmental consequences. People are often at risk of exposure to styrene because it is used in a wide variety of consumer items, including plastics, electrical and thermal insulation, fibreglass, pipes, vehicle parts, and carpet backing [3]. In addition, styrene can contaminate food items packaged using polystyrene materials [4]. In terms of styrene absorption, inhalation has been found to be a more efficient route than skin contact, as supported by previous studies [5,6]. Following a sequence of metabolic processes within the organism, styrene is converted into PGA, which is subsequently eliminated from the body through urinary excretion [6]. The International Agency for Research on Cancer (IARC) has classified styrene as a Group 2B carcinogen, indicating that it is a substance that has the potential to cause cancer in individuals [7,8,9]. The protection of workers’ health from any adverse effects that may be caused by exposure to chemicals on the job is the primary focus of occupational toxicology. According to the guidelines established by ACGIH, the maximum recommended occupational exposure dose of PGA should not exceed 250 mg/L, and the detection of the concentrations of PGA and MA in either urine or serum is the most effective analytical technique [10,11].

Chemical analysis necessitates the use of precise equipment and clean samples that are completely isolated from interfering substances [12]. Sample preparation is an essential step in the analysis of the chemicals present in biological samples. Numerous analytical methodologies have been reported for the determination of PGA, including High Performance Liquid Chromatography−mass spectrometry (HPLC–MS), gas chromatography–mass spectrometry (GC–MS), voltammetry, reagent-free ion chromatography, and multi-pumping flow systems. Furthermore, the predominant technique utilised for the separation of solvents from urine specimens is SPE, which is preferred mainly owing to its simplicity, efficiency in terms of time, and minimal solvent consumption [13,14]. In a recent study, Peng et al. (2021) created an ultra-stable metal–organic framework (MOF) using terbium (Tb–MOF 1). They then used this framework to fabricate a luminescence film (1/PLA) by combining it with polylactic acid (PLA). The resulting film was used as a sensor for rapid detection of PGA. The researchers discovered that the sensor exhibits notable benefits in terms of effective detection, with a limit of detection (LOD) of 1.05 × 10^−4^ mg/mL, and a fast response time, taking less than 10 s, to detect PGA in urine. Significantly, the extraction percentage was within the range of 98–104%, and the RSD% was between 2.15 and 3.63%. High stability, exceptional interference prevention capabilities, and superior recyclability were cited as the benefits of the Tb–MOF sensor [14].

Although SPE sorbents have many benefits, they may not always exhibit high selectivity for metabolites. Interferents may cause problems in the analysis if they are eluted with the target analytes and retained via the sorbent. Molecularly imprinted polymers (MIPs) can be utilised as SPE sorbents to address these concerns, thereby conferring improved selectivity and enrichment to the desired analyte compared to other analogous compounds. Molecular imprinting is widely acknowledged for its remarkable capability to create custom-designed recognition sites that preserve the specific attributes of template molecules, including their shape, size, and functional groups. This unique feature allows molecularly imprinted polymers (MIPs) to exhibit high selectivity and affinity for target analytes. Moreover, MIPs have extensive utility in developing chemosensors, providing the added advantage of collective responses that enhance sensitivity by “amplifying” signals compared to single molecular systems [15,16]. This amalgamation of features makes molecular imprinting a powerful and versatile technique, with diverse applications in various fields.

This innovative approach is founded on the utilisation of MISPE, which is a combination of MIP and SPE techniques [17,18,19]. Upon completion of the polymerisation process, the extraction of the target analyte from the MIP was achieved using appropriate organic solvents. Monomers that possess identical analytes or structurally similar chemicals have the potential to bind to the three-dimensional cavity created by chemical interactions [13,20]. Noncovalent strategies are frequently employed for the synthesis of MIPs used for environmental analytes, including pure organic solvents and biological fluids. This approach is preferred due to its simplicity, ease of template molecule removal, and the availability of numerous functional monomers in the commercial market [20,21,22]. Bulk preparation techniques have been predominantly employed for the synthesis of most reported MIPs, owing to their numerous advantageous features. The capabilities encompass the proficiency of producing MIPs of superior quality while minimising waste generation and avoiding the need for expensive or complex machinery. Subsequently, the mass polymer monolith undergoes a process of fragmentation, comminution, and classification, yielding nonuniformly configured particles, primarily within the size range of 25–100 µm [23,24]. The MISPE system comprises multiple modes. Fundamental operational states encompass both offline and online modalities. There are no apparent distinctions between offline MISPE protocols and other SPE processes. The procedure comprises conditioning of the MISPE cartridge, subsequent loading of the sample, removal of any potential interference through washing, and ultimately, elution of the analyte. The elution phase in the MISPE process is a critical step and a significant improvement factor for a favourable analyte recovery rate. Typically, polar solvents are employed in conjunction with minor amounts of alkaline or acidic additives, while using minimal amounts of solvent [25].

The selection of an appropriate functional monomer is crucial because it plays a key role in determining the stability of the formed complex during the polymerisation process and the subsequent selective interaction of the molecularly imprinted polymer (MIP) with the target molecule. In the case of most functional monomers, selective retention of analytes on the polymer is achieved through hydrogen bonding or ionic interactions, depending on the solvent and pH of the sample being processed. Response surface methodology (RSM) offers a valuable approach for determining the optimal template/monomer/crosslinking agent molar ratio, reducing the need for extensive experimental MIP synthesis and analysis [20,24,26]. MIPs consist of several components, including a matrix, monomer, crosslinker, initiator, and porogen. It is crucial that the monomer interacts with the matrix to form specific donor–receptor complexes necessary for successful polymerisation. For acidic templates such as PGA, the use of basic functional monomers, such as 4-VP, is recommended [27]. More recently, Qronfla et al. [28] reported a novel MISPE incorporating an imprinting polymer, including 4-VP as a basic functional monomer, using mandalic acid as a template, EGDMA as a cross-linking agent, AIBN as an initiator, and ACN as a porogen solvent at a standard molar ratio of 1:4:20 through bulk polymerisation. Through our comprehensive analysis of the available literature, we found no previous study that utilised the molecularly imprinted solid-phase extraction (MISPE) technique for the extraction of the urine metabolite phenylglyoxylic acid (PGA) as a biomarker of exposure to styrene.

Optimising the extraction of template molecules from molecularly imprinted polymers (MIPs) remains a challenging task because variations in pH, sorbent mass, and elution flow rate can affect the adsorption capacity and recovery during the desorption phase [28,29,30]. Therefore, it is crucial to control these influential variables to ensure optimal process efficiency. By identifying the variables that maximise the extraction of the target molecule, it is possible to overcome the limitations of matrix extraction under practical test conditions, thereby enabling better quantification of metabolites in human urine samples [7,22,28]. Response surface methodology (RSM) is commonly employed to assess parameters and investigate interactive effects [31,32,33,34,35,36,37]. However, few studies have explored the optimal extraction of metabolite-related compounds from urine. Among these rare studies, Heravizadeh et al. [30] successfully demonstrated a selective extraction process for the herbicide metribuzin from urine samples by combining polymer nanoparticles and response surface methodology. Consequently, utilising optimal conditions for extracting urinary biomarkers via molecularly imprinted polymers presents a promising and efficient approach for assessing human exposure to aromatic compounds and associated risks.

The primary objective of this study was to prepare a molecularly imprinted polymer, 4-VPMIP, for selective solid-phase extraction (SPE) of phenylglyoxylic acid (PGA). The synthesised MIP was characterised by Fourier transform infrared (FT-IR) spectroscopy and scanning electron microscopy (SEM). To optimise the adsorption and desorption procedures, various processing parameters, such as pH, elution volume, flow rate, sorbent mass, and elution volume were systematically investigated using a central composite design with response surface methodology (RSM). Quantification and performance assessment of the method were performed using high-performance liquid chromatography with a diode array detector (HPLC–DAD). The response variables were evaluated by determining the optimal settings for the experimental factors and a desirability index was generated.

## 2. Materials and Methods

### 2.1. Chemicals

All chemicals and solvents employed in the proposed research were analytical reagents, and ultrapure water was used throughout the experiments. The solvents used in this study were provided by reliable suppliers. Phenylglyoxylic acid (PGA) and hippuric acid (HA) were obtained from Fisher Scientific Co. (Hampton, NH, USA), Thermo Scientific Co. (Shanghai, China), and Fisher Scientific Co. (Hampton, NH, USA), respectively. The purity of these solvents was reported to be 97%, 99%, and 99%, respectively, with a weight of 100 g for HA. Hydrochloric acid (HCl), EGDMA (98%), ethyl ether (C4H10O, 95%), and anhydrous sodium sulfate (Na₂SO_4_, 99% purity) were supplied by Merck KGaA (Darmstadt, Germany). The reagents used in this study were purchased from Sigma-Aldrich Co., Ltd. (St. Loui, MO, USA), and included 4-VP (95% purity, with 100 ppm hydroquinone as an inhibitor), AIBN solution, and MAA (99% purity). HPLC-grade acetonitrile (ACN) was supplied by Fisher Scientific Co. HPLC-grade acetonitrile (ACN) and methanol (MeOH) were supplied by Hampton (NH, USA). Glacial acetic acid (AA, CH3COOH, 100%) was obtained from PanReac AppliChem (Castellar del Vallès, Spain). Prolabec (Laval, QC, Canada) provided acetone. A steel cylinder of nitrogen gas with a volume of 10 L and purity of 99.999%, identified as NG01RTN02, was obtained from ASG in KSA. The cylinder was used to eliminate oxygen gas. The Millipore purification system at the Industrial Waste Treatment Lab (KAU) of the Environmental Sciences Department provided ultrapure water. The system and MPK01 filter were manufactured by Millipore (Fontenay-sous-Bois, France). The inhibitors were removed from EGDMA by vacuum distillation. The inhibitors in 4-VP were removed by passing them through a simple Al_2_O_3_ column, and AIBN was recrystallised from MeOH before use. One of the research groups generously provided urine for this experiment. Urine samples were collected in the morning and placed in sterile containers before placing them in a freezer at −20 °C to preserve the samples for future analysis.

### 2.2. Analytical Instruments

High-performance liquid chromatography (HPLC) with a diode array detector (DAD) from Agilent Technologies 1200 series (Santa Clara, CA, USA) was used for the analytical separation. A digital ultrasonic cleaner (JPS-24AD, 3 L, Moscow, Russia) was used to disperse the mixtures and remove oxygen from the solution. The polymerisation reactions were conducted in an oil bath. For better separation during chromatographic analysis, a Fisher Scientific Centrifuge (accuSpinTM Micro, Schwerte, Germany) with 24 tube positions was employed. The IR spectra of the polymer particles were investigated using IRAffinty-1 Spectroscopy SHIMADZU (Kyoto, Japan) in the range of 4000–400 cm^−1^. Thermogravimetric analysis (TGA) was conducted using an SDTQ600 thermal analyser (PerkinElmer Pyris Diamond, Norwalk, USA) under a controlled nitrogen atmosphere. The heating rate was set at 10 °C/min. Scanning electron microscopy (SEM) using a Quanta 250 (Waltham, MA, USA) was used to examine the morphology of the polymer particles. To wash PGA from the MIPs during the elution step, MAX Empty SPE Cartridges Solid-Phase Extraction 3 mL with two frits from JVLAB (Shanghai, China) was used. Grinding of MIP particles to a minimum granularity of 0.1 um was achieved using a 0.4 L laboratory pulveriser ball mill small planetary ball grinding mill machine (DECO, Hunan Yueyang, China) with compatible grinding jars (ball, PTFE, Teflon) and balls (ZrO_2_; Zirconium oxide). Particle sizes ≤ 38 µm were obtained using a standard test sieve (55 × 28 mm) of a 400-mesh stainless steel screen cell strainer (Shijiazhuang, China) with a handle. The Environmental Science Department’s Food Safety and Quality Lab utilised an SPE vacuum manifold (CHROMABOND^®^) with 18 positions for PGA extraction during the elution process. Additionally, the Water Pollution Lab employed a dryer oven (Heraeus, Hanan, Germany) and an analytical balance (Mettler Toledo AL204, Columbus, OH, USA) to dry the polymers and weigh the exact masses of the MIP components.

### 2.3. Preparation of Imprinted Polymer PGA–MIP and NIP

**Figure 1:**
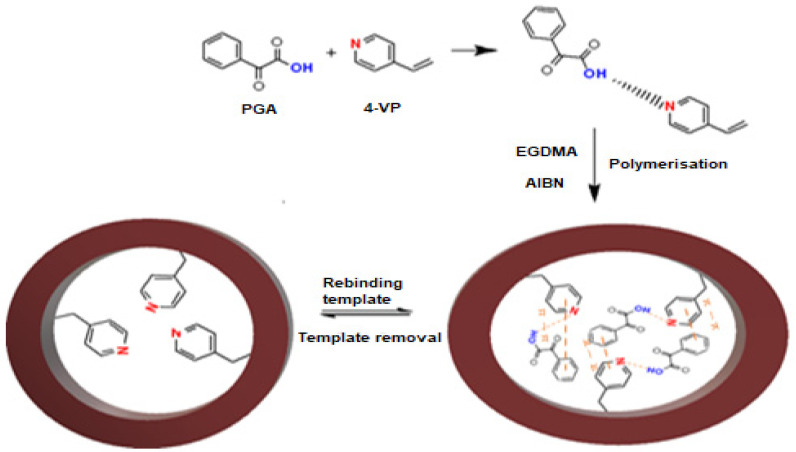
Schematic illustration of PGA-MIP synthesis steps and PGA removal from MIP binding sites.

The method for the preparation of core–shell molecular imprinted polymers (MIP) was adopted from a method similar to that described by Murad Qronfla et al. [28]. First, 1 mmol of PGA (template) was mixed with 3 mL of ACN and 4 mmol of 4-VP (functional monomer) in a 10 mL glass test tube. The test tube was subsequently placed in an ultrasonic bath (30 min) at room temperature. Subsequently, the test tube was expeditiously sealed, and the solution was purged with nitrogen gas to remove any dissolved oxygen, as described in [38]. Subsequently, 20 mmol of EGDMA and 478 µL of AIBN were injected. Sonification of the solution was continued for an additional twenty minutes. Following the deoxygenation process, the reaction mixture was heated in an oil bath at 60 °C for 24 h, with purging with N_2_ gas. Figure 1 depicts a flowchart of the MIP creation procedure.

**Figure 2:**
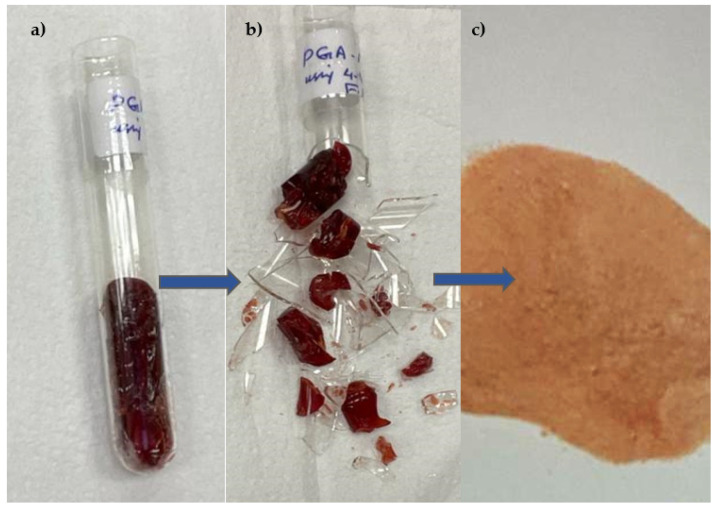
The generated MIP. (**a**) Bulk polymerisation. (**b**) Crushing the polymer vial for grinding. (**c**) The 4-VPMIP powder of PGA was in its complete state.

The polymer was then dried in an oven at 60 °C for 24 h. Subsequently, the polymer was crushed and sieved at ambient temperature Figure 2, resulting in particles measuring 38 µm or less. The test sieve used for this process had a mesh size of 40 μm. Non-imprinted polymer (NIP) was synthesised in a comparable fashion; however, the template molecule was not present. Table 1 displays the procedures employed to generate the MIP and NIP for the PGA.

The template elution process is crucial for MIP generation. The objective was to selectively extract PGA molecules from MIP, resulting in the formation of cavities with dimensions, morphology, and chemical functionalities complementary to those of PGA. If PGA molecules remain within the MIP, the number of available cavities for rebinding would decrease, ultimately resulting in a reduction in their efficacy. A washing solution under the optimal conditions of pH, methanol, elution volume, and flow rate were used to eliminate PGA and all the residues left during the synthesis. The MIP was dried at 60 °C for 6 h and then stored at room temperature.

**Figure 3:**
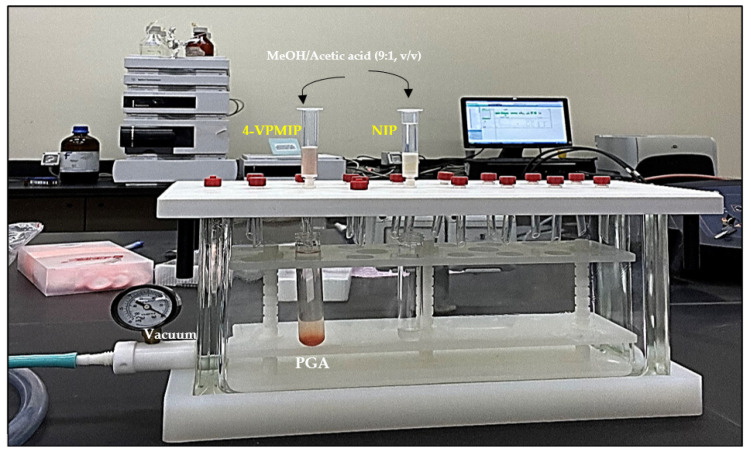
MIP or NIP wash process.

In the experimental procedure, a 3 mL SPE cartridge was utilised to perform the elution process. The cartridge was packed with the optimal sorbent mass of MIP or NIP between the two polyethylene frits. The compressed powder specimen was extracted through the immobile phase under optimal elution conditions using an SPE vacuum manifold at a controlled extraction speed and sample flow, as depicted in Figure 3. Subsequently, the specimen was injected into a high-performance liquid chromatography-diode array detector at a wavelength of 225 nm to quantify PGA. The procedure was iterated four times, covering the conditioning phase in which PGA became undetectable.

### 2.4. Polymer Characterisation

FT-IR spectroscopy was used to characterise the structures of the 4-VPMIP and 4-VPNIP particles. Spectral analysis was conducted within the wavenumber range of 500–4000 cm^−1^, with a spectral resolution of 2 cm^−1^. SEM was used to examine the morphology of the polymer surfaces. Dry polymer specimens were plated with a light layer of gold before being used in SEM studies.

### 2.5. Adsorption Capacities (MIP/NIP)

Adsorption capacities were studied using the following procedure: 10 mg of the polymer (MIP or NIP) was added to 10 mL, with initial concentrations of PGA ranging from 2.5 to 50 mg. L^−1^ spiked in the human urine sample. After ultrasonication for 2 h, the supernatant was collected by centrifugation at 4000 rpm. The concentration of the supernatant was measured using HPLC–DAD. The amount of PGA bound by the MIP or NIP was calculated using the following equation:

(1) $$Q_{e}=\frac{\left(C_{0}-C_{e}\right)\times V}{w}$$

where, *Q_e_* (mg mg^−1^) is the adsorption capacity by MIP or NIP, *C*_0_ (mg mL^−1^) is the initial concentration of PGA, *C_e_* (mg mL^−1^) is the concentration of the supernatant liquid, *V* (mL) is the volume of PGA solution, *m* (mg) is the mass of MIP or NIP.

### 2.6. HPLC–DAD-Analysis Conditions

A high-performance liquid chromatography (HPLC) method was employed for the analysis, which involved the utilisation of a binary pump, autosampler, column compartment, and diode array detector (DAD) that monitored the effluent for PGA at a wavelength of 225 nm. Optimisation of the analytical technique for PGA determination was conducted according to previous studies [11,28,39]. The optimal mobile phase composition for HPLC was determined to be a mixture of ACN, ultrapure water, and acetic acid in specific proportions. The mixture was composed of three components in volumetric proportions of 60:39.5:0.5. A C18 column with dimensions of 250 × 4 mm and a particle size of 5 μm was used in the experiment. The column was maintained at a constant temperature of 22 °C. The experimental parameters were configured as follows: injection volume, 20 µL; flow rate, 0.7 mL/min, injection volume, 5 μL.

### 2.7. Analytical Performance of MIP Extraction

The HPLC–DAD method was used to assess the linear range (LR) and relative standard deviation (%RSD) for different concentrations of PGA. PGA peaks were identified by analysing the retention time and peak area at the appropriate HPLC wavelength. The relationship between the peak area and PGA concentration was analysed using linear least-squares regression. A standard curve was generated, and a regression equation was applied to estimate PGA concentrations in diverse human urine samples [28,39]. Additionally, the analytical performance of the method was evaluated by determining the limits of detection (LOD) and limits of quantification (LOQ), as described by the following equations:

(2) $$LOD=\frac{3\times s_{d}}{m}$$

(3) $$LOQ=\frac{10\times s_{d}}{m}$$

where *s_d_* is the standard deviation of ten replicates of the lowest concentration standard sample prepared in water and methanol mixture and *m* is the slope of the calibration curve.

### 2.8. Optimisation of PGA Adsorption and Desorption by MIP Using Experimental Design Approach

Optimisation of the adsorption and desorption of phenylglyoxylic acid (PGA) through MIP–SPE involved the selection of four key parameters: sample pH, sorbent mass, sample flow rate, sample volume, and flow rate of the eluting solvent. These parameters were selected based on previous studies and preliminary experimental tests [28]. The response surface statistical method was chosen to design optimisation experiments and modulate the process of adsorption and recycling of PGA by MIP-SPE. The selected operational ranges of the variables are presented in Table 2. A Central composite design (CCD) (24 + star) of orthogonal type with nine centre points was established via a combination of Statgraphics Centurion XVI software package version 16 and MATLAB R2019a version wherein the rotatability of the design was ensured with α = 1.86792.

Based on the proposed design (CCD) and backward algorithm for model building, 18 experiments with different combinations of four factors were performed. The retention (adsorption) and extraction (desorption) of PGA through MIP (4-VP)SPE were selected as the responses. A second-order model was used in response surface methodology. The equation is expressed as follows:

(4) $$\hat{Y}=\beta_{0}+\sum_{i=1}^{4}\beta_{ii}X_{i}^{2}+\sum_{i=1}^{3}\sum_{j=i+1}^{4}\beta_{ij}X_{i}X_{j}+\varepsilon$$

where $\hat{Y}$ denotes the predicted response of the process; *β*_0,_ *β_i_*, *β_ii_*, *β_ij_* are the regression coefficients of intercept, linear, quadratic, and interactive terms, respectively. *X_i_*, *X_j_* are levels of the coded levels of the factors (independent or control variables) and ε is the statistical error.

In the designed experiments, a 6 mL capacity SPE cartridge containing MIP-4VP in the range of 15–30 mg (factor x_2_ in Table 2) was placed between two frits. The MIP(4-VP)-SPE column was sequentially conditioned using 1 mL methanol and distilled water at a flow rate of 1 mL/min. Subsequently, a PGA solution with a concentration of 50 mg/L was passed through the cartridge at a specified pH (factor x_1_) and flow rate (factor x_3_), with a variable volume between 3 and 6 mL (factor x_4_). The resulting solution was injected into an HPLC system for analysis. The adsorption efficiency of the MIP was determined using the following equation, where C_i_ and C_f_ represent the concentrations of PGA in the solution before and after adsorption, respectively:

(5) $$Adsorption\;efficiency\left(\%\right)=\frac{\left(C_{i}-C_{f}\right)}{C_{i}}\times 100$$

To optimise the extraction of PGA from MISPE and minimise the number of tests, the quantities of PGA loaded onto the column and defined during the optimisation of the adsorption phase were subjected to an elution phase, considering the operational desorption factors (Table 2). The experiments were carried out in 18 series designed using the same software (STATGRAPHICS XVI version 16 and MATLAB R2019 a version). The percentage of extracted PGA was calculated using the following equation:

(6) $$Extraction\;efficiency\left(\%\right)=\frac{C_{ext}}{C_{ads}}\times 100$$

where C_ext_ and C_ads_ are the PGA concentration extracted and adsorbed, respectively.

### 2.9. Selectivity Studies

To perform selectivity studies, the desorbed polymer containing the template was combined with equal volumes of solutions containing PGA (template) and hippuric acid (HA) metabolites found in urine, both at the same concentration. The extent of binding was determined using HPLC–DAD and the differences in binding between the compounds were compared. For the experiment, either 20 mg of MIP or NIP was added to 5 mL of binary mixtures containing PGA/HA at a concentration of 50 mg/L under optimal adsorption conditions. The levels of free analyte and other urine metabolites in the supernatant were analysed using HPLC–DAD. The equations used to calculate the partition coefficient (*K_d_*, mL.g^−1^) [28,40], imprinting factor (*IF*), and selectivity coefficient (*α*) were as follows:

(7) $$K_{d}=\frac{C_{0}-C_{e}}{C_{0}}\times \frac{V_{s(mL)}}{\left(Mass\;of\;MIP\;or\;NIP(g)\right)}$$

(8) $$IF=\frac{K_{d}(MIP)}{K_{d}(NIP)}$$

(9) $$\alpha =\frac{{IF}_{PGA}}{{IF}_{HA}}$$

where *C*_0_ and *Ce* are the initial and equilibrium concentrations of the analyte, respectively, and Vs is the volume of the solution. *IF_PGA_* and *IF_HA_* were the imprinting factors for the template molecule *PGA* and *HA*.

### 2.10. Reusability Studies

The reusability of the imprinted polymers was assessed using the following procedure: 20 mg of the polymer was added to a solution containing 40 mg/L PGA in 5 mL of a blank urine sample and stirred for an hour. The mixture was then centrifuged at 3500 rpm for 10 min to separate the polymer and supernatant. The adsorbent polymers were then washed with a solution of MeOH–acetic acid (9:1, *v*/*v*) until PGA was completely removed. HPLC–DAD analysis was performed to determine the extraction and desorption efficiencies of PGA. The polymers were vacuum-dried overnight at 60 °C and subsequently reused for PGA adsorption. This sorption–desorption cycle was repeated until PGA was no longer detectable.

## 3. Results and Discussion

### 3.1. Characterisation of Molecular Imprinted Polymer

**Figure 4:**
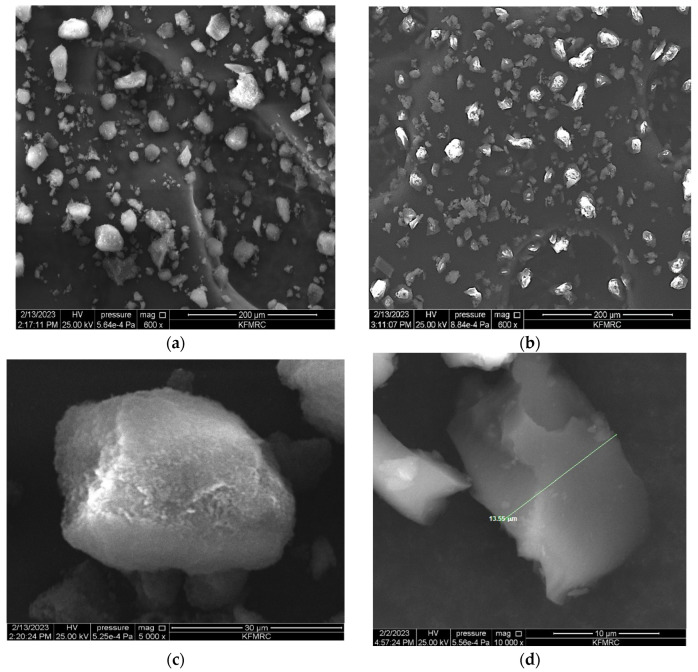

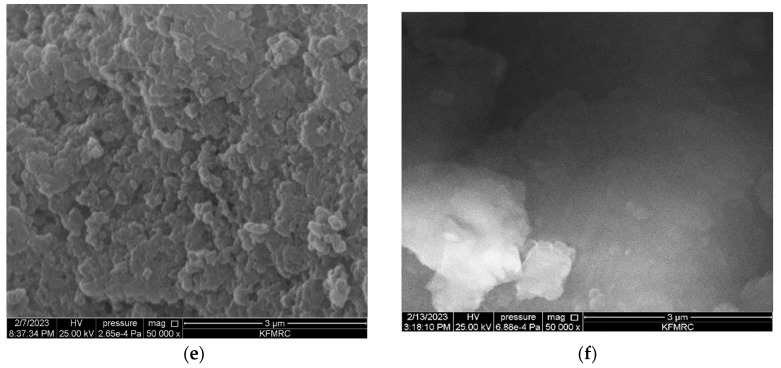
SEM images at different magnitudes of (**a**,**c**,**e**) 4VPMIP (**b**,**d**,**f**) 4VPNIP.

**Figure 5:**
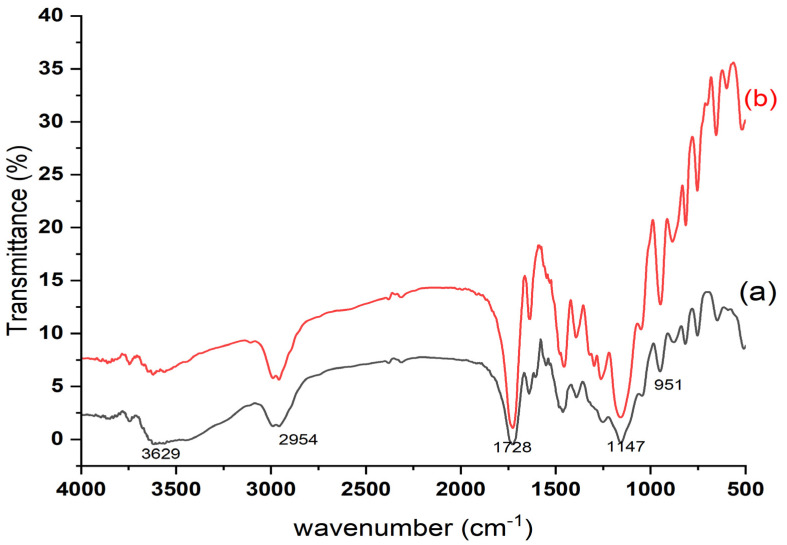
FT-IR spectra of MIP before (a) and after washing (b) PGA removal.

SEM analysis is a critical technique in morphological research because it enables the determination of the form and dimensions of the polymer particles. The present study employed SEM analysis to observe the generation of irregular particles in the micrometre range. The observed irregularities were attributed to the utilisation of bulk polymerisation during the synthesis of polymer particles. These tiny particles significantly increased the surface contact between the template and the MIP, which eventually absorbed a significant amount of PGA. The difference in particle size between the MIP and NIP particles, which is shown in Figure 4, is caused by the participation of the template molecule in the imprinting process. The creation and expansion of the particle surfaces increased because of the addition of PGA to the reaction solution, allowing MIP particles to grow in size. In addition, the elution of PGA chemicals in the MIPs left a wide variety of voids, making the MIP surfaces rougher and more porous than the NIP surfaces. This evidence demonstrates the successful elimination of PGA from MIP, resulting in the incorporation of its imprint onto the polymer matrix through the utilisation of PGA as the molecular template and 4-VP as the functional monomer in the bulk polymerisation process for the preparation of MIP particles. The adsorption of analytes (PGA) was notably higher in MIP than in NIP, owing to the presence of cavities in the PGA–MIP particles, resulting in a larger surface area than that of NIP. Differences in the SEM images indicate the presence of PGA molecules during bulk polymerisation, resulting in a different polymeric evolution in terms of structure, even if imprinted holes could not be seen because of the small size of the PGA molecule. It appears that all the synthesised polymers exhibit a rigid nature. The presence of distinct morphological characteristics suggested that MIP and NIP were successfully formed [17,40,41,42,43]. When polymeric materials are combined, heated, or exposed to light, their molecular structures or contents may change. Figure 5 shows the IR spectra used to determine the functional groups in the MIP before (a) and after elution (b), as well as in the NIP (c), and to ensure the interaction among PGA, 4-VP, and EGDMA. The presence of PGA as an analyte in the MIP matrix material causes the IR spectra of MIPs with PGA (before washing) to be semi-similar to those of NIP, while the spectra of MIP (after washing) showed compositions and absorption peaks that were very similar to those of NIP, demonstrating that all MA were eliminated after the extraction phase [44]. The chemical structures of MIP and NIP were identical despite their morphological variance. Thus, the findings obtained from the FT-IR and SEM analyses indicate that the MIPs were successfully synthesised.

### 3.2. FT-IR Spectroscopy

Polymeric materials can undergo changes in their molecular structure or composition when subjected to various factors such as mixing, heating, or exposure to light. These alterations can occur rapidly, within seconds, or may require several hours to complete. Figure 5 illustrates the IR spectra utilised to determine the presence of functional groups in the molecularly imprinted polymers (MIPs) before (a) and after elution (b) to confirm the interaction between PGA, 4-VP, and EGDMA. The presence of PGA as an analyte within the matrix material of the MIPs is responsible for the observed IR spectra of MIPs with PGA (prior to the wash), whereas the spectra of the MIPs (after the wash) exhibit similar compositions and absorption peaks, indicating the complete elimination of all PGA molecules during the extraction phase [41]. In all polymers, the polymerisation process was confirmed by the presence of C-O-C and C=O bonds of EGDMA, as evidenced by the bands at 1147 cm^−1^ and 1728 cm^−1^, respectively. Can Zhou et al. have also observed characteristic peaks of EGDMA at 1718 cm^−1^ for C=O and 1637 cm^−1^ for C=C stretching vibrations [40]. In contrast, the IR spectra of the MIPs (before the wash) and MIPs (after the wash) demonstrated stronger C=O stretching vibrations at 1728 cm^−1^ and weaker C=C stretching vibrations at 1633 cm^−1^. This indicates effective cross-linking of EGDMA within the polymer monoliths. Furthermore, the C-H stretching vibration mode of the aliphatic compound was observed in the range of 2954 cm^−1^ to 2995 cm^−1^. Two peaks indicative of 4-VP were observed: a stretching vibration peak at 1633 cm^−1^ due to the C=N functional group in the aromatic ring and a very weak signal at 1521 cm^−1^ due to the C=C functional group in the vinyl ring. The presence of the template, which engages in hydrogen bonding with 4-VP (spectra (b)), likely contributed to the relatively broad peak observed at 3629 cm^−1^ compared to the infrared spectra of the MIPs after washing [42]. These results demonstrate the successful polymerisation of 4-VP into MIPs. Consequently, the FT-IR spectra strongly suggested the successful synthesis of MIPs.

### 3.3. Thermal Properties

**Figure 6:**
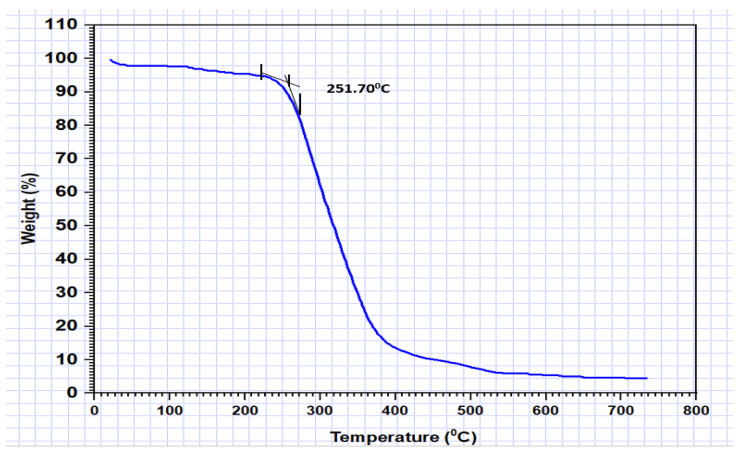
Thermogravimetric analysis of a PGA-imprinted polymer in a nitrogen atmosphere at a heating rate of 10 °C/min.

Thermogravimetric analysis (TGA) revealed the excellent thermal stability of the crosslinked polymers. Thermogravimetric analysis (TGA) was conducted by heating the samples under a controlled nitrogen atmosphere at 10 °C/min. As shown in Figure 6, the weight loss of the PGA-imprinted polymer is plotted against temperature. The inset in the figure indicates that decomposition of the polymer occurs at 251.70 °C, demonstrating its robust thermal stability.

### 3.4. Adsorption Capacity

As anticipated, the MIP exhibited a higher adsorption capacity than the NIP, which was attributed to its specific imprinted sites on the polymer for the target analyte [17]. Based on this finding, MIP was chosen exclusively to optimise the extraction procedure. Adsorption studies were conducted to explore the adsorption behaviour of both MIP and NIP. The equilibrium adsorption data for PGA onto the polymers were analysed using Langmuir [45] and Freundlich [45] isotherms, employing nonlinear Equations (10) and (11).

(10) $$\mathrm{Langmuir}:\;q=\frac{q_{m}\;k_{L}C_{e}}{1+k_{L}C_{e}}$$

where *q_e_*, *q_m_*, *k_L_* and *C_e_* are amount of PGA adsorbed, maximum adsorption capacity, Langmuir constant and concentration of trenbolone at equilibrium, respectively.

(11) $$\mathrm{Freundlich}:\;q_{e}=K_{F}C_{e}^{\frac{1}{n}}$$

where *K_F_*, *C_e_* and *n* are the measure of adsorption capacity, equilibrium concentration and indicator of adsorption effectiveness, respectively.

**Figure 7:**
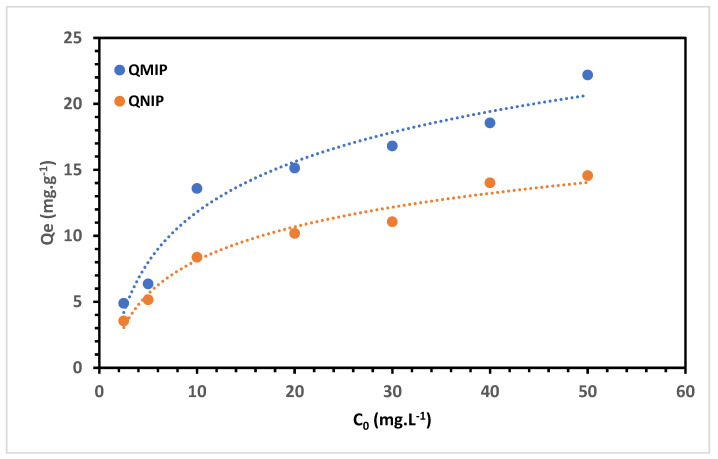
Adsorption capacity studies for the MIP and NIP.

The adsoption capacities of MIP and NIP are presented in Figure 7.

The adsorption data acquired for PGA on the MIP demonstrated a better fit to the Langmuir isotherm, with a correlation coefficient of 0.9645, surpassing the R^2^ value of 0.7865 obtained for the Freundlich isotherm. This outcome indicates that the adsorption of PGA onto the MIP is uniform and suggests monolayer adsorption [46,47]. The maximum adsorption capacity calculated using the Langmuir isotherm was determined to be 27.5 mg g^−1^.

### 3.5. Optimisation of MIP(4-VP)SPE Procedure by Experimental Design Approach

The results obtained for the adsorption and desorption of PGA using the MIP(4-VP)SPE procedure are presented in Table 3, respectively, for all CCD runs. The response values ranged from 72.02% to 99.69% of the extraction and from 3.02% to 100.00% of the retention.

To determine the significant factors, interaction effects, and optimal extraction conditions, the optimisation results were analysed using analysis of variance (ANOVA), response surface methodology, and desirability function. Statistical estimators obtained from the analysis of variance (ANOVA) were used to evaluate the adequacy of the reduced quadratic models (Table 4 and Table 5). The F-value indicator, which measures the variance of the data, was employed to determine the statistical significance of the model. Based on the findings presented in Table 4 and Table 5, the reported F-values for the selected models in the adsorption and desorption steps deviate significantly from unity, indicating high-level and reliable predictions based on the empirical data. Additionally, the low *p*-values in both phases indicated the statistical validity of the models in predicting the response. The quality of fit for the polynomial model equation was expressed by the coefficient of determination (R^2^) and adjusted coefficient of determination (R^2^_adj_) in ANOVA. R^2^ represents the proportion of the variation in the response, which can be explained by the predictors in the model. In this study, both the adsorption and desorption models exhibited desirable R^2^ values close to 1. Moreover, the predicted R^2^ values aligned with the adjusted coefficient of determination (R^2^_adj_), indicating that the factors influencing the efficiency of the MIP-4VPSPE procedure were appropriately selected. Importantly, the value for the lack of fit in the ANOVA tables is higher than 0.05, suggesting that the model is acceptable for the given data at the chosen confidence level.

The ANOVA in Table 4 partitioned the variability in adsorption into separate pieces for each effect. It then tested the statistical significance of each effect by comparing its mean square against an estimate of the experimental error. In this case, six effects have a *p*-value of less than 0.05, indicating that they are significantly different from zero at the 95.0% confidence level. The R-squared statistic indicates that the adjusted model explains 99.5018% of the variability in adsorption. The adjusted R-squared statistic, which is more suitable for comparing models with different numbers of independent variables, is 97.1771%. The standard error of the estimate shows that the standard deviation of the residuals was 4.86827. The mean absolute error (MAE) of 1.77003 was the average value of the residuals. The Durbin–Watson (DW) statistic tests the residuals to determine if there is any significant correlation based on the order in which they occur in the data file. Because the *p*-value is greater than 5.0%, there is no indication of serial autocorrelation in the residuals at the 5.0% significance level.

In this case, for the desorption step, eight effects had a *p*-value of less than 0.05, indicating that they were significantly different from zero at the 95.0% confidence level. The R-squared statistic indicates that the adjusted model explains 99.5209% of the variability in extraction. The adjusted R-squared statistic, which is more appropriate for comparing models with different numbers of independent variables, is 97.2853%. The standard error of the estimate shows that the standard deviation of the residuals was 1.55107. The mean absolute error (MAE) of 0.507682 was the average value of the residuals. As the *p*-value was less than 5.0%, there was an indication of possible serial correlation at the 5.0% significance level.

In the subsequent phase, employing the multivariate regression method, empirical models were established for the prediction of PGA in terms of coded factors, based on the significant regression coefficients (*p* < 0.05) obtained from the reduced quadratic models. These models were utilised to estimate the PGA adsorption and desorption efficiencies through the MIP(4-VP)SPE procedure. The experimental adsorption and desorption data were fitted to second-order models and are expressed as follows:

Adsorption step:

Retention (%) = −120.965 + 12.1265 × x_1_ − 3.811 × x_1_^2^ + 0.450551 × x_1_ × x_2_ + 2.29442 × x_1_× x_4_ − 0.144035 × x_2_^2^ − 32.0105 × x_3_^2^ (12)

Desorption step:

Extraction (%) = 18.9891 + 9.15336 × x_1_ + 1.1907 × x_2_ + 11.93 × x_3_ + 11.6443 × x_4_ − 1.54598 × x_1_^2^ + 0.169351 × x_1_ × x_2_+ 0.497964 × x_1_ × x_4_ − 0.0535623 × x_2_^2^ − 3.48447 × x_3_^2^ − 1.55733 × x_4_^2^ (13)

where, x_1_, x_2_, x_3_, and x_4_ are the values of four independent variables (pH, sorbent mass, elution flow rate, elution volume).

**Figure 8:**
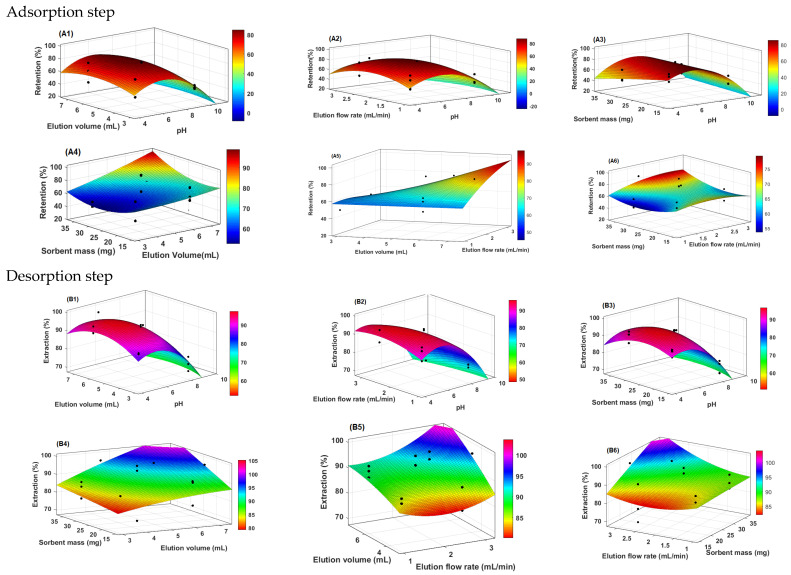
Response surface methodology and showing the interaction between the independent factors (**A1/B1**) pH and elution volume, (**A2/B2**) pH and elution flow rate, (**A3/B3**) pH and sorbent mass, (**A4/B4**) Elution volume and sorbent mass, (**A5/B5**) Elution volume and elution flow rate, (**A6/B6**) Elution flow rate and sorbent mass.

The response surface models were visually represented through response surface methodology plots, which showed the interaction effects of the operational variables on the response factors (retention and extraction of PGA). Figure 8 shows 3D response surface plots illustrating these relationships.

Optimising the pH in solid-phase extraction (SPE) procedures is crucial for effective intermolecular interactions, such as ionic interactions, hydrogen bonding, and hydrophobic interactions between the analytes and the molecularly imprinted polymer (MIP). The results found in this study confirm previous research [48,49]. In addition, its interactive relationship with the other three independent variables also had an important effect on the adsorption and desorption efficiencies, as shown in the Figure 8A1–A3/B1–B3. As can be seen, for both the adsorption and desorption processes, increasing the pH of the sample to levels above 6 decreases the adsorption and desorption of phenylglyoxylic acid. At pH values close to the pKa value of approximately 3.2, most of the phenylglyoxylic acid is in its protonated, non-ionised form which promotes hydrogen binding by the MIP. However, as the pH increases and becomes higher than the pKa, a greater proportion of phenylglyoxylic acid exists in its anionic form which reduces the ability of the sorbent’s 4-vinylpyridine functional groups to effectively bind to the analyte of interest (PGA). The combined effect of the flow rate (sample and solvent) and other factors (sorbent mass, pH, and elution volume) revealed that better adsorption and desorption of PGA were obtained when the flow rate was between 1 mL/min and 2 mL/min (Figure 8A2/B2,A5/B5,A6/B6). Increasing the flow rate of the sample negatively affected the sorbent adsorption efficiency. This can be attributed to several factors, including inadequate contact time between the sample and sorbent, decreased mass transfer of the analyte molecules to the sorbent, and heightened competition for binding sites on the sorbent surface [50]. The shorter contact times at higher flow rates limit the opportunity for effective interaction between the analytes and sorbent, leading to a decrease in the adsorption efficiency [51]. Additionally, inadequate mass transfer and increased competition further contributed to the reduction in adsorption efficiency. The interactive effect between sorbent mass and elution volume shows that high extraction (desorption) of PGA was obtained when the eluent volumes were greater than 6 mL (Figure 8B4). Contrary to what is observed in the adsorption step (Figure 8A4), a high elution volume can decrease the adsorption of the analyte on the sorbent owing to several factors. First, the larger volume of the solvent used for elution can dilute the analyte concentration, resulting in a lower concentration available for adsorption onto the sorbent. Second, the weaker elution power of the solvent at high volumes may reduce its ability to effectively compete with the sorbent for binding sites, leading to a lower elution efficiency. Lastly, the increased elution volume can disrupt the interaction between the analyte and sorbent, causing the analyte molecules to be washed away more easily and reducing their affinity for the sorbent surface. These results are in perfect agreement with those of Sangai et al. [52]. By examining the combined effect of sorbent mass and other factors such as elution flow rate, pH, and elution volume, it was observed that improved adsorption occurred as the sorbent mass increased within the range of 15–30 mg (Figure 8 A3/A4/A6). This indicated that a higher mass of the polymer provided an increased number of binding sites for the template, resulting in the quantitative retention of PGA. Therefore, increased sorbent mass enhances the adsorption capacity and improves the efficiency of PGA retention.

**Figure 9:**
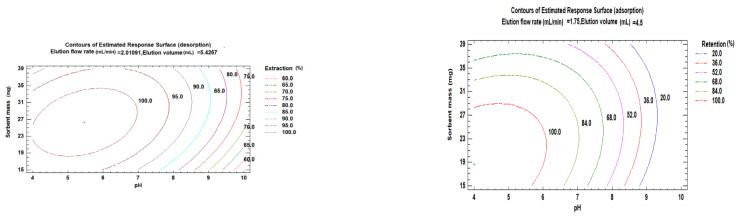
Contours of estimated response surface and showing the region that maximises adsorption and desorption for a fixed elution flow rate and elution volume.

The combination of factor levels, which maximises retention (adsorption) and extraction (desorption) in one of the regions on which the optimisation will be performed, can be determined from one value or several factors, as shown in Figure 9. The estimated response surface contours illustrate the region that maximises the adsorption and desorption for a fixed elution flow rate and elution volume. These results from the contours of the estimated response surfaces show that high adsorption of PGA is reached at an estimated pH value equal to 4.0 for a fixed rate and volume of elution at values 1.75 mL and 4.5 mL, respectively. A similar trend was observed for the best PGA desorption, which was obtained at an estimated pH of 5.46 for flow rate and volume values of 2.0 mL/min and 5.4 mL, respectively.

**Figure 10:**
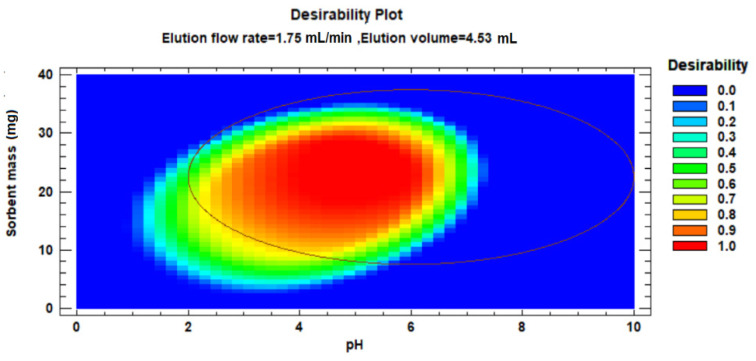
Desirability profiles and predicted values for factors affecting the adsorption of PGA.

The desirability profile and predicted values (Figure 10) were utilised to determine the optimal conditions based on the desirability of each factor. A desirability value of 1 was chosen as the target, serving as a guide for estimating the conditions necessary to achieve optimum signal enhancement.

Finally, based on the obtained regression models, analysis of variance (ANOVA) for the response surface quadratic model, optimum values of selected parameters with respect to the desirability plot, and consideration of the desired efficiency. The optimum operating conditions for the adsorption process were determined as follows: sample pH 4, sorbent mass, 18.70 mg, sample flow rate, 1.87 mL/min and 4.0 mL of elution sample for the adsorption process. Based on the same approach, the optimum operating conditions for the adsorption process were determined as follows: sample pH 5.68, sorbent mass, 26.29 mg, sample flow rate, 2 mL/min and 5.4 mL of elution solvent for the adsorption process.

### 3.6. Adsorptive Selectivity

To assess the selective adsorption capacity of 4-VPMIP (molecularly imprinted polymer) for phenylglyoxylic acid (PGA), a binary mixed solution containing PGA and hippuric acid (HA), both of which are urine metabolites, was prepared to serve as competitive adsorption molecules. Distribution coefficient (Kd, mL). g^−1^), imprinting factor (IF), and relative selectivity coefficient (α) were calculated using Equations (3)–(5), respectively, as described in the Experimental section. The results of the cross-selectivity assessment of MIP are presented in Table 3, demonstrating a successful imprinting effect on the polymer and its selectivity. The greater selectivity observed for PGA can be attributed to the following factors. First, the distribution coefficients of PGA in the 4-VPMIP were higher than those of the interfering HA, with values of 272.57 and 89.33, respectively. The higher Kd value for PGA confirms the enhanced selectivity of MIP compared to that of the non-imprinted polymer (NIP). Second, the imprinting factor (IF) values for PGA were higher for the MIP than for the interferents, indicating a higher degree of selectivity and affinity of the MIP for the target molecule PGA. Finally, the relative selectivity coefficient (α) values for the binary mixtures of PGA/HA were greater than 1, specifically 2.78, indicating the excellent affinity of 4-VPMIP for PGA adsorption. (Table 6).

### 3.7. Analytical Performance and Applicability of MIP(-4VP)SPE to Real Urine Samples

Analytical performance was evaluated using the limits of detection (LOD) and quantification (LOQ), reproducibility, and selectivity of the method. The calibration curves of PGA and HA were established using five concentrations 5, 25, 50, 125 and 250 mg.L^−1^, prepared by serial dilution of the stock solution in spiked urine. Calibration curves were constructed by plotting the peak area ratios of PGA and HA against their concentrations. The regression equations for PGA and HA were y = (5.492 ± 0.025)x + (0.013 ± 0.006), (R^2^ = 0.9995) and y = (2.422 ± 0.018)x + (0.037 ± 0.014), (R^2^ = 0.9978), respectively. The slopes and intercepts are presented as ± standard error. The LODs and LOQs for each compound were determined based on calibration curves (Table 7).

**Figure 11:**
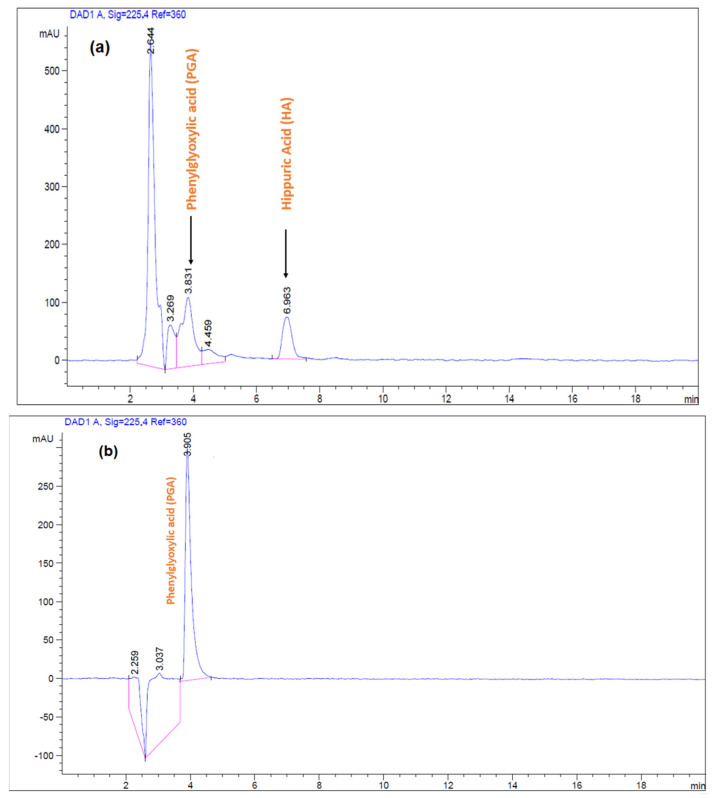
HPLC chromatograms of a urine sample spiked with a standard mixture of HA and PGA (**a**), Eluate of desorption of PGA from a spiked urine sample after MIP(-4VP)PES extraction (**b**).

The selectivity of the analytical method was evaluated by comparing the chromatograms acquired for the extracted urine samples spiked with a standard mixture of HA and PGA with those for the eluate of desorption of PGA from a spiked urine sample after MIP(-4VP)PES extraction. The obtained results (Figure 11) indicated a non-significant effect of matrix interference on the sensitivity and selectivity of the prepared MIP(4-VP). Furthermore, to assess the reproducibility of the proposed method, ten consecutive experiments were performed for a single PGA concentration (100 mg.L^−1^) over three different days. A relative standard deviation (RSD) of 3.84% was obtained which shows the satisfactory performance of the optimised MIP(4-VP)SPE method for PGA determination. Urine samples provided by one of our research teams were spiked with PGA at 5, 50, and 125 mg.L^−1^. The optimised MIP(4-VP)SPE method was then applied to analyse the PGA. As shown in Table 8, the recovery of PGA from spiked samples with different concentrations was sufficiently acceptable, demonstrating the reliable, selective, and accurate performance of the synthesised MIP (4-VP) in cleaning up complex samples.

### 3.8. Reusability of SPE-4-VPMIP

**Figure 12:**
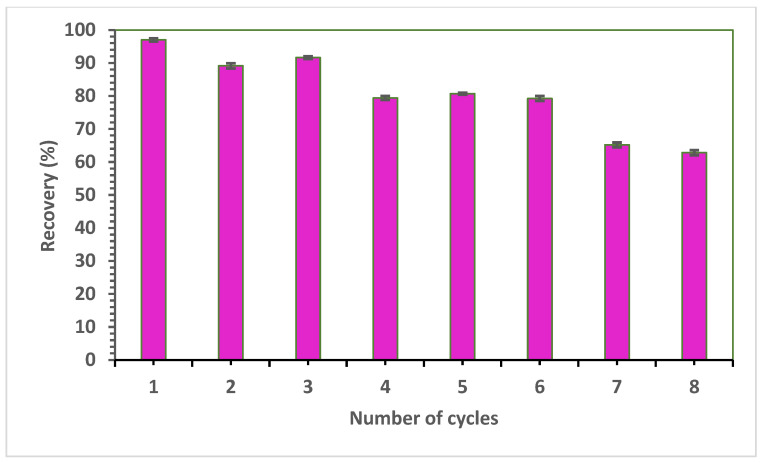
PGA percent recovery across multiple reuse cycles of the MIP (*n* = 3).

The reusability of the 4-VPMIP cartridges was assessed by performing a series of preconcentration procedures followed by elution, polymer washing with the eluent, and vacuum drying for reuse after each cycle. Eight complete cycles were performed to evaluate the reusability of the polymer. Figure 12 illustrates the adsorption efficiency of SPE4-VPMIP over eight consecutive adsorption–desorption cycles using an initial PGA concentration of 100 mg/L. This test played a crucial role in demonstrating the potential reusability of SPE4-VPMIP. The results revealed that SPE4-VPMIP exhibited consistent efficiency over the first three adsorption–desorption cycles, with the removal efficiency maintaining 90% of the initial efficiency (Figure 12). However, in Cycle 7, the efficiency decreased to 65%. This reduction in uptake can be attributed to the progressive saturation of active sites within 4-VPMIP, which inhibits further template uptake.

## 4. Conclusions

In this study, we present a novel sample preparation technique that combines molecularly imprinted polymers (MIP) and solid-phase extraction (SPE) for the analysis of PGA in human urine. MIPs were synthesised using a bulk polymerisation protocol and a non-covalent approach utilising PGA as the template, 4-VP as the functional monomer, and EGDMA as the cross-linking agent. The characterisation of the synthesised MIP using FTIR spectroscopy and SEM confirmed its suitable morphology and physicochemical properties as an effective sorbent for SPE. The presence of permanently porous microparticles further facilitated the efficient removal and preconcentration of PGA from complex samples. The cross-linked polymers demonstrated excellent thermal stability, as evident from thermogravimetric analysis (TGA), with decomposition occurring at 251.70 °C. By employing a central composite design (CCD) approach, the adsorption and desorption procedures were optimised, resulting in the determination of optimal operating conditions for both processes. Under the optimised conditions, the selectivity parameters exhibited a higher degree of selectivity and affinity for PGA, with a relative selectivity coefficient (α) of 2.79 against hippuric acid. The developed analytical methods show promise for the detection of target analytes in urine samples. PGA adsorption exhibited a preference for the Langmuir isotherm, suggesting a monolayer mechanism for the adsorption process. Furthermore, MIP demonstrated recyclability for up to three cycles, yielding recoveries above 90%. This method, based on an experimental design and a modern modelling approach, allows for the optimisation of experimental conditions, estimation of interactions between factors, and attainment of more satisfactory results compared to a one-at-a-time approach. Overall, this technique holds significant potential for the analysis of PGA as a biomarker of styrene exposure in complex matrices, such as urine.

## Figures and Tables

**Table 1:** Synthesis protocol of 4-VPMIP and 4-VPNIP for PGA.

Polymer	Template	Functional Monomer	Cross Linker	Mole Ratio	Progen	Initiator
MIP	PGA	4-VP	EGDMA	1:4:20	ACN	AIBN
NIP	-	4-VP	EGDMA	1:4:20	ACN	AIBN

**Table 2:** Operational range of input variables for experimental design.

MIP(4-VP)SPE Phase	Input Variables	Unit	Symbol	Levels	
				Lower	Upper
Adsorption Desorption	pH	-	x_1_	4.0	9.0
	Sorbent mass	mg	x_2_	15.0	30.0
	Elution flow rate	mL/min	x_3_	1.0	2.5
	Elution solvent volume	mL	x_4_	3.0	6.0

**Table 3:** Central composite design matrix observed response in desorption step (Extraction %) and observed response in adsorption step (Retention %).

Run	X_1_	X_2_	X_3_	X_4_	Extraction (%) (Mean ± SD)	Retention (%) (Mean ± SD)
1	4	15	1	3	88.41 ±0.24	81.01 ± 0.76
2	4	30	1	3	86.45 ± 0.03	79.04 ± 0.85
3	8	30	1	3	75.89 ± 0.02	27.02 ± 0.13
4	8	15	2.5	3	72.02 ± 0.53	23.00 ± 0.15
5	4	30	2.5	3	88.99 ± 0.25	81.05 ± 0.42
6	8	30	2.5	3	79.85 ± 0.84	26.08 ± 0.18
7	4	15	1	6	92.12 ± 0.17	85.12 ± 0.06
8	8	15	1	6	77.50 ± 0.95	29.03 ± 0.12
9	4	30	1	6	94.03. ± 0.23	64.04 ± 0.73
10	8	30	1	6	89.46 ± 0.25	41.08 ± 0.06
11	4	15	2.5	6	92.80 ± 0.09	92.06 ± 0.42
12	8	15	2.5	6	79.23 ± 0.05	42.02 ± 0.77
13	4	30	2.5	6	95.80 ± 0.24	62.16 ± 0.10
14	9.73	22.5	1.75	4.5	69.02 ± 0.36	3.02 ± 0.04
15	6	36.5	1.75	4.5	96.04 ± 0.42	72.16 ± 0.19
16	6	22.5	3.15	4.5	97.22 ± 0.09	48.03 ± 0.05
17	6	22.5	1.75	7.3	96.71 ± 0.04	92.00 ± 0.19
18	6	22.5	1.75	4.5	99.69 ± 0.17	100.00 ± 1.05

SD standard deviation for *n* = 3.

**Table 4:** Analysis of variance (ANOVA) for the response surface quadratic model (retention:adsorption step).

Source	Sum of Squares	*df*	Mean Square	F-Ratio	*p*-Value
x_1_: pH	6621.71	1	6621.71	279.40	0.0005
x_2_: Sorbent mass	36.7358	1	36.7358	1.55	0.3015
x_3_: Elution flow rate	147.332	1	147.332	6.22	0.0882
x_4_: Elution volume	67.8588	1	67.8588	2.86	0.1892
x_1_x_1_	1537.69	1	1537.69	64.88	0.0040
x_1_x_2_	438.174	1	438.174	18.49	0.0231
x_1_x_3_	1.25352	1	1.25352	0.05	0.8329
x_1_x_4_	454.533	1	454.533	19.18	0.0220
x_2_x_2_	447.886	1	447.886	18.90	0.0225
x_2_x_3_	134.314	1	134.314	5.67	0.0976
x_2_x_4_	99.793	1	99.793	4.21	0.1325
x_3_x_3_	2145.37	1	2145.37	90.52	0.0025
x_3_x_4_	17.9202	1	17.9202	0.76	0.4485
x_4_x_4_	151.498	1	151.498	6.39	0.0856
Total error	71.1002	3	23.7001		
Total (corr.)	14,272.5	17			
Multiple R^2^ = 0.9950; Adjusted R^2^ = 0.9717					
Standard Error of Est. = 4.86827; Mean absolute error = 1.77003					
Durbin–Watson statistic = 1.74455 (*p* = 0.1104); Lag 1 residual autocorrelation = 0.107803					

*df* degree of freedom.

**Table 5:** Analysis of variance (ANOVA) for the response surface quadratic model (extraction:desorption step).

Source	Sum of Squares	*df*	Mean Square	F-Ratio	*p*-Value
x_1_: pH	328.097	1	328.097	136.38	0.0013
x_2_: Sorbent mass	87.7088	1	87.7088	36.46	0.0091
x_3_: Elution flow rate	21.0238	1	21.0238	8.74	0.0597
x_4_: Elution volume	184.642	1	184.642	76.75	0.0031
x_1_x_1_	253.047	1	253.047	105.18	0.0020
x_1_x_2_	61.9063	1	61.9063	25.73	0.0148
x_1_x_3_	1.91072	1	1.91072	0.79	0.4385
x_1_x_4_	21.4099	1	21.4099	8.90	0.0584
x_2_x_2_	61.9367	1	61.9367	25.74	0.0148
x_2_x_3_	0.863509	1	0.863509	0.36	0.5913
x_2_x_4_	17.1837	1	17.1837	7.14	0.0755
x_3_x_3_	25.4209	1	25.4209	10.57	0.0475
x_3_x_4_	0.369892	1	0.369892	0.15	0.7212
x_4_x_4_	83.7748	1	83.7748	34.82	0.0097
Total error	7.21747	3	2.40582		
Total (corr.)	1506.59	17			
Multiple R^2^ = 0.9952; Adjusted R^2^ = 0.9728					
Standard Error of Est. = 1.55107; Mean absolute error = 0.507682					
Durbin-Watson statistic = 1.29337 (*p* = 0.0224); Lag 1 residual autocorrelation = 0.335221					

*df* degree of freedom.

**Table 6:** Selectivity parameters of 4-VPMIP (distribution coefficient (Kd), imprinting factor (IF) and relative selectivity coefficient (α)).

Target and Metabolites Solution	Polymer	Kd (mL/g)	IF	α(IF_PGA_/I_FHA_)	
PGA	MIP	272.57	2.34		2.79
	NIP	116.17			
HA	MIP	89.33	0.84		
	NIP	106.37			

**Table 7:** Detection and quantification limits.

Metabolite	LOD (mg.L^−1^)	LOQ (mg.L^−1^)
PGA	0.5	1.6
HA	3.4	11.4

**Table 8:** Recovery of PGA from spiked urine samples under optimal condition.

Spiked Urine (mg.L^−1^)	Recovery (%)	*RSD* (%) (*n* = 3)
5	97.32 ± 4.29	4.61
50	98.78 ± 3.08	3.72
125	99.06 ± 3.25	3.51

*RSD* relative standard deviation.
